# Lactopeptine

**Published:** 1879-06

**Authors:** 


### Lactopeptine.

Lactopeptine, a combination of Pepsin, Sugar of Milk, Pancreatine, Ptyaline and Lactic and Hydrochloric Acids is now offered to the profession, and is highly recommended in the impaired digestion of old people, and in obstinate cases of diarrhœa of young children.

